# Sodium dichloroacetate improves migration ability by suppressing LPS-induced inflammation in HTR-8/SVneo cells via the TLR4/NF-κB pathway

**DOI:** 10.22038/IJBMS.2023.68252.14902

**Published:** 2024

**Authors:** Cheng Lu, Zhen-Wei Zhou, Yu Jiang, Jianzhong Li, Jia-Bei He, Chuan Zhang, Alex F Chen, Xia Tao, Cheng Peng, He-Hui Xie

**Affiliations:** 1 School of Public Health and Hongqiao International Institute of Medicine, Shanghai Jiao Tong University School of Medicine, Shanghai 200025, China; 2 Department of Pharmacy, Second Affiliated Hospital of Naval Medical University, Shanghai 200003, China; 3 Department of Biochemical Pharmacy, School of Pharmacy, Naval Medical University, Shanghai 200433, China; 4 School of Medicine, Shanghai University, Shanghai 200444, China; 5 Institute for Developmental and Regenerative Cardiovascular Medicine, Xinhua Hospital, Shanghai Jiao Tong University School of Medicine, Shanghai 200092, China; # These authors contributed eqully to this work

**Keywords:** HTR-8/SVneo cells, Inflammation, Migration, Sodium dichloroacetate, Toll-like receptor-4

## Abstract

**Objective(s):**

Inadequate cytotrophoblast migration and invasion are speculated to result in preeclampsia, which is a pro-inflammatory condition. Sodium dichloroacetate (DCA) exerts anti-inflammatory actions. Thus,we sought to investigate the effect of DCA on the migration function of the lipopolysaccharide (LPS)-stimulated human-trophoblast-derived cell line (HTR-8/SVneo).

**Materials and Methods:**

HTR-8/SVneo cells were treated with LPS to suppress cell migration. Cell migration was examined by both scratch wound healing assay and transwell migration assay. Western blotting was used to analyze the expression levels of toll-like receptor-4 (TLR4), nuclear factor-κB (NF-κB), TNF-α, IL-1β, and IL-6 in the cells.

**Results:**

DCA reversed LPS-induced inhibition of migration in HTR-8/SVneo cells. Furthermore, DCA significantly suppressed LPS-induced activation of TLR4, phosphorylation of NF-κB (p65), translocation of p65 into the nucleus, and the production of pro-inflammatory cytokines (TNF-α, IL-1β, and IL-6). Treatment with inhibitors of TLR4 signal transduction (CLI095 or MD2-TLR-4-IN-1) reduced LPS-induced overexpression of pro-inflammatory cytokines, and a synergistic effect was found between TLR4 inhibitors and DCA in HTR-8/SVneo cells.

**Conclusion:**

DCA improved trophoblast cell migration function by suppressing LPS-induced inflammation, at least in part, via the TLR4/NF-κB signaling pathway. This result indicates that DCA might be a potential therapeutic candidate for human pregnancy-related complications associated with trophoblast disorder.

## Introduction

Preeclampsia (PE) is a complex pregnancy disorder that affects 2% to 8% of pregnancies worldwide. Maternal spiral artery remodeling and poor placental implantation are associated with this condition (1-3). During pregnancy, one of the prominent factors for proper implantation and placentation is migration of extravillous trophoblast (EVT) cells into spiral arterioles. Only after invasion can trophoblast cells successfully degrade and migrate through the extracellular matrix to interact closely with the endothelial cells of the uterine spiral arteries and further replace them (4, 5). Insufficient trophoblastic invasion of the decidua and spiral arteries is considered to be the first stage of PE development (6, 7). For researchers in reproductive medicine, the question of how to promote trophoblast cell function is of particular interest.

Though the pathophysiology of PE remains ill-defined, there is a large body of evidence that shows the inflammatory reaction is significantly enhanced in the pathophysiology of PE, involving several pro-inflammatory factors. Migration and invasion of trophoblast cells are affected by those cytokines (8-12). As a treatment for congenital lactic acidosis and other diseases, sodium dichloroacetate (DCA) is a potent and safe agent (13, 14). Recent studies have found that DCA may exert some anti-inflammatory actions (15-18). A study in the rheumatoid arthritis mouse model has shown that DCA administration alleviates the development of arthritis in female mice (17). Thus, it can be logically speculated that DCA could modulate trophoblast cell migration function by inhibiting the inflammatory response.

We performed lipopolysaccharide (LPS) treatment on trophoblast cells to suppress cell migration ability in this investigation. Toll-like receptor-4 (TLR4) can be activated by LPS, and TLR4 activation can recruit NF-κB and increase several chemokines and inflammatory cytokines synthesis, for example, tumor necrosis factor-alpha (TNF-α), interleukin-6 (IL-6), and interleukin-1β (IL-1β) (19, 20). As a family member of pathogen-related molecule pattern recognition molecules, TLR4 has an integral part in the initiation and acceleration of inflammation (21). As a downstream of TLR4-mediated signaling, the NF-κB signaling pathway has a critical effect in amplifying the inflammatory response by increasing the expression of diverse pro-inflammatory cytokine genes (22). Therefore, we aimed to investigate in this study whether DCA could ameliorate LPS-induced HTR-8/SVneo cell migration ability impairment and examine if DCA showed anti-inflammatory roles through suppression of the TLR4/NF-κB pathway.

## Materials and Methods

***Reagents***

Trypsin from Gibco (USA) and RPMI 1640 were purchased from Hyclone ( USA). Aspirin (ASP), lipopolysaccharide (LPS), and Fetal bovine serum (FBS) were purchased from Sigma (USA) together with DCA and Hoechst 33258. Antibodies against NF-κB p65 (p65), IL-1β, IL-6, phospho-NF-κB p65 (p-p65), TNF-α, TLR4, and β-actin were purchased from Abcam (UK). Antibodies against TNF-α, TLR4, IL-6, IL-1β, NF-κB p65 (p65), phospho-NF-κB p65 (p-p65), and β-actin were purchased from Abcam (UK). Antibodies against TLR4 extracellular binding domain (MD2-TLR-4-IN-1) and intracellular binding domain (CLI095) were purchased from MedchemExpress (USA).

***Cell culture***

The International Peace Maternity & Child Health Hospital of the China Welfare Institute (China) provided human chorionic trophoblast cells (HTR-8/SVneo). Cells were plated at 5 × 10^5^ cells/well on a 6-well plate and cultured overnight in RPMI 1640 medium supplemented with 10% (v/v) fetal bovine serum (FBS) under 5% CO_2_ at 37 °C (23).

***Cell viability assay***

The viability of cells after treatment with different doses was determined using the Cell Counting Kit-8 (Dojindo, Japan) to detect the cytotoxicity of LPS. In 96-well plates, 5000 cells per well were cultivated for 24 to 48 hr. LPS were added to different groups at 0.1, 0.2, 0.5, or 1 μg/ml final concentrations when 90% confluence had been reached. A replacement medium containing 10% CCK-8 was added after 24 hr and incubated for an additional hour. With a microplate reader, we measured optical density at 450 nm (24). 

***LPS treatment***

**Figure 1:**
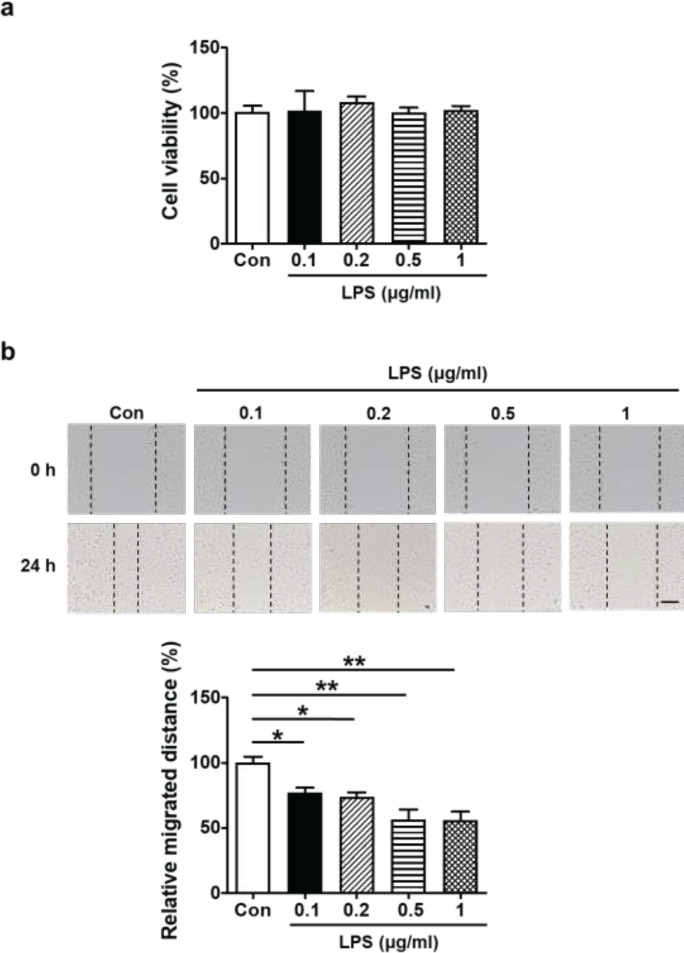
Effect of LPS on cells viability and migration in HTR-8/SVneo cells

Cells were cultured using HTR-8/SVneo cells according to Section 2.2, using LPS to suppress cell migration. After treating the cells with different concentrations of LPS (0.1, 0.2, 0.5, or 1 µg/ml) for 24 hr, the culture medium was changed to RPMI 1640 with 1% (v/v) FBS. We found that LPS (0.1, 0.2, 0.5, or 1 µg/ml) significantly inhibited HTR-8/SVneo cell migration. What is more, LPS-induced cell migration dysfunction was dose-dependent, and reached maximal values at 0.5 µg/ml (Figure 1b). Taking together our results as well as other researchers’ study (19), we selected the LPS concentration (0.5 µg/ml) for the continuation of this study.

***DCA treatment***

Cell culture of HTR-8/SVneo was performed in accordance with previous section cells were incubated respectively with 1% (v/v) FBS RPMI 1640 culture medium containing vehicle, LPS (0.5 µg/ml), LPS (0.5 µg/ml) + DCA (0.01, 0.05, or 0.25 mmol/l), and LPS (0.5 µg/ml) + ASP (10 µg/ml) for 24 hr. The cells were then used for migration function assay (both transwell migration assay and scratch wound healing assay) and western blotting analysis.

***Scratch wound healing assay***

**Figure 2:**
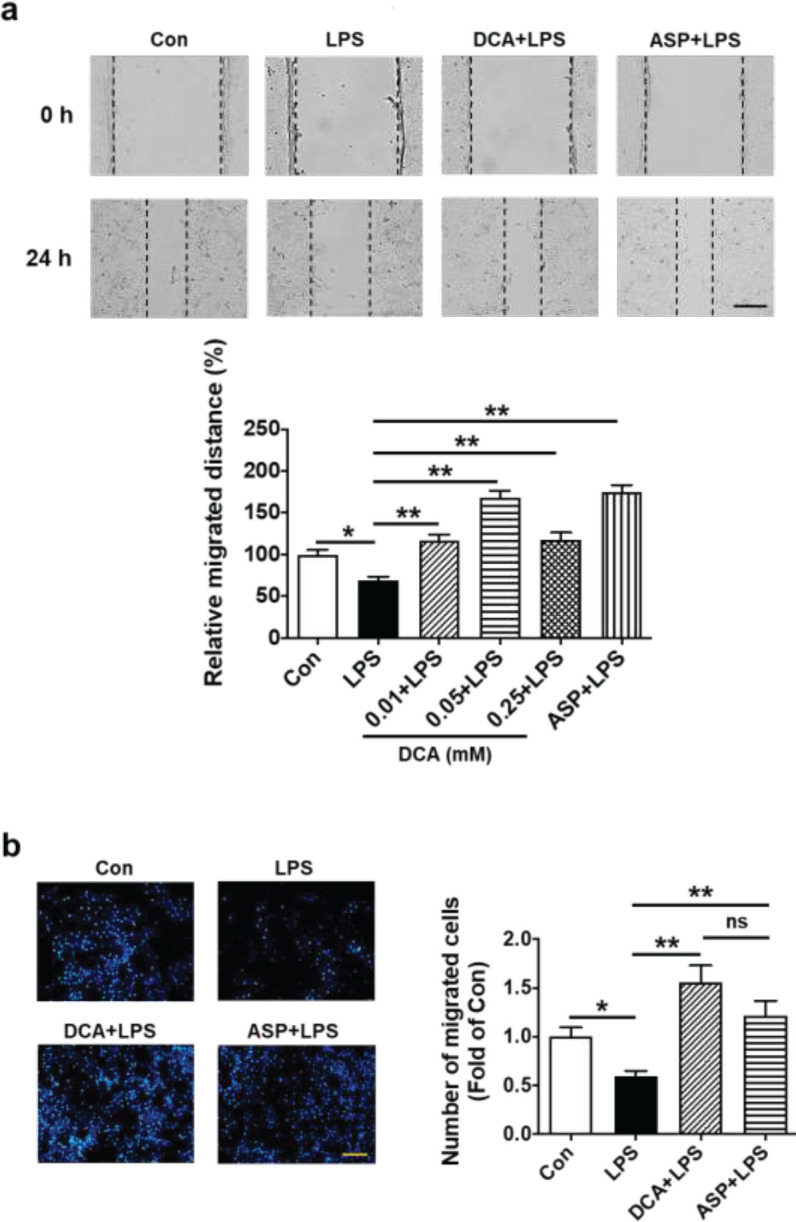
DCA promoted HTR-8/SVneo cell migration function impaired by LPS

Cell migration capability was assessed using a scratch wound healing assay. That is, cells were cultured in 6-well plates at a density of 1.0 × 10^5^, and then monolayers of cells were scraped with a pipette tip and washed with PBS. The cells were subjected to treatment conditions as stated in Sections 2.4 and 2.5. Photographs were captured utilizing an inverted microscope at two distinct time points, namely 0 hr and 24 hr. Measurements were taken at various locations to determine the width of the scratch (25, 26). It was shown that DCA (0.01, 0.05, or 0.25 mmol/l) significantly improve HTR-8/SVneo cell migration when stimulated by LPS, and the effect of DCA was comparable with that of ASP (10 μg/ml) at the concentration of 0.05 mmol/l (Figure 2a). Thus, the concentration of DCA (0.05 mmol/l) was used in the following experiments of the present work.

***Transwell migration assay***

By using the transwell migration assay, this assay assessed the migration potential of HTR-8/SVneo cells. A total of four different cell treatments were employed: vehicle, LPS (0.5 µg/ml), LPS (0.5 µg/ml) + DCA (0.05 mmol/l), and LPS (0.5 µg/ml l) + ASP (10 µg/ml) for 24 hr, following trypsinization, the target concentration of the cell suspension was 1.0 × 10^5^ cells/ml. 100 µl of serum-free medium was added to the supra-chamber and 10% FBS medium was added to the infra-chamber. Incubation for 24 hr was followed by staining with Hoechst 33258 for 15 min and fixing with 2% (v/v) paraformaldehyde. Five fields of each sample (magnification × 100) were counted randomly for the number of migrated cells, and the average of the five different regions was calculated (27-29). In all experiments, blinding and randomization were applied.

***RT-PCR and quantitative RT-PCR (qRT-PCR)***

Real-time PCR analysis was performed to assess the expression of TNF-α, TLR-4, IL-6, and IL-1β (30, 31). 

RNA isolation and cDNA preparation RNA was extracted from cells using the RNeasy Mini Kit (Qiagen). Prior to use, optical density was tested at 260 and 280 nm to define the concentration and purity of the RNA. Each sample’s total RNA (1 g) was used for reverse transcription. DNA synthesized with FastKing RT kit and gDNase (Tianjian).

TNF-α, TLR-4, IL-6, and IL-1β mRNA expression was examined using quantitative real-time PCR tests using the SuperReal PreMix Plus SYBR Green kit (TIANGEN). The expression of β-actin mRNA was measured as the internal control. PCR cycling conditions included pre-heating for one cycle at 95 °C for 15 min, amplification for 40 cycles at 95 °C for 10 sec and 60 °C for 32 sec, and chilling to 40 °C. Molecular levels of mRNA were quantified and normalized against mRNA levels of β-actin. Each control group›s averaged and normalized mRNA levels were expressed as 1.0. 

The following primers were used for quantitative RT-PCR:

TLR4

FWD: GACTGGGTAAGGAATGAGCTAG,

REV: ACCTTTCGGCTTTTATGGAAAC

TNF-α,

FWD: TGGCGTGGAGCTGAGAGATAACC, 

REV: CGATGCGGCTGATGGTGTGG

IL-1β, 

FWD: GCCAGTGAAATGATGGCTTATT, 

REV: AGGAGCACTTCATCTGTTTAGG 

IL-6, 

FWD: CACTGGTCTTTTGGAGTTTGAG

REV: GGACTTTTGTACTCATCTGCAC

β-actin, 

FWD: CTACCTCATGAAGATCCTGACC, 

REV: CACAGCTTCTCTTTGATGTCAC

***Western blotting***

Cultured trophoblast cells, 1.5 × 10^6^ cells/50 µl, were homogenized by adding protease inhibitor (KC-440, KangChen), and total protein lysates were prepared using centrifugation at 12,000 g for 10 min at 4 °C. The Pierce BCA assay kit (Thermo Fisher) was used to determine protein concentrations, and samples with the same amount of protein were submitted to 10% (v/v) SDS/PAGE. After protein transfer to nitrocellulose membranes, the membranes were blocked with 5% (w/v) skimmed milk (Bio-Rad) and incubated overnight at 4 °C with a series of primary antibodies, namely TLR4 (1:1000), p-p65 (1:600), p65 (1:600), IL-1β (1:1000), IL-6 (1:1000), TNF-α (1:500), and β-actin (1:5000). The secondary antibody used was IRDye® 800CW goat anti-rabbit/mouse IgG secondary antibody (1:10,000). NIH Image J software was used to quantify bands visualized with Odyssey Imager 1.1 software (Li-Cor).

***Cellular NF-*** ***κB*** ***p65 translocation assay***

For NF-κB translocation experiments, HTR-8/SVneo cells (3 × 10^5^ /well) were fixed after 1 hr DCA (0.05 mmol/L) treatment, and then simultaneously to LPS (0.5 µg/ml) for 20 min. Cells were fixed in 2% (v/v) paraformaldehyde and permeabilized with 0.3% (v/v) triton-X100 in PBS for 15 min, blocked with bovine serum albumin (BSA) / PBS for 1 hr and incubated overnight with rabbit anti-NF-κB p65 (1:200, Abcam), followed by biotinylated anti-rabbit IgG for 1 hr. Nuclei were stained with Hoechst 33258 for 15 min. A fluorescence microscope was used to count the number of NF-κB-positive cells in three different sections (32). In all experiments, blinding and randomization were applied.

***Inhibitor treatment***

To further examine whether DCA exerted anti-inflammatory roles by inhibiting the TLR4/NF-κB pathways, antibodies against TLR4 intracellular binding domain (CLI-095) and extracellular binding domain (MD2-TLR4-IN-1) were added at final concentrations of 10 µmol/L. Briefly, HTR-8/SVneo cells were pretreated with or without CLI-095 or MD2-TLR-4-IN-1 for 1 hr before incubation with LPS or DCA for 24 hr (33, 34). Then, the cells were harvested by trypsin digestion and centrifugation for western blotting analysis.

***Statistical analysis***

The data were shown as mean ± SEM. The statistical significance of group differences was ascertained using one-way ANOVA, and a Turkey *post hoc* analysis was then carried out. *P*-values < 0.05 were regarded as significant in statistics. 

## Results

***Different concentrations of LPS inhibit HTR-8/SVneo cell migration***

To explore how LPS affects the migration of HTR-8/SVneo cells and avoids their cytotoxicity, we conducted cell viability assays and scratch wound healing assays. Following a 24 hr treatment with different concentrations of LPS (0.1, 0.2, 0.5, or 1 μg/ml), it was discovered that cell viability remained unaffected (Figure 1a), Based on the outcomes of the cell viability experiments, the scratch assay was used to assess how different LPS dosages affected the migratory function of HTR-8/SVneo cells. It was found that LPS-induced cell migration dysfunction was dose-dependent and reached maximal values at 0.5 µg/ml (*P<0.05, **P<0.01; Figure 1b). On the basis of these data and other researchers’ study (19), the 0.5 µg/ml dose was therefore selected for subsequent experiments. 

***DCA improves LPS-inhibited HTR-8/SVneo cell migration***

Scratch wound healing assay and transwell migration assays were used to analyze the impact of DCA on the migration function of LPS-stimulated HTR-8/SVneo cells. It was found that DCA could significantly improve cell migration distance (**P<0.01; Figure 2a) and migration number (**P<0.01; Figure 2b). The effect of DCA was comparable with that of ASP (10 μg/ml) in promoting HTR-8/SVneo cell migration at the concentration of 0.05 mmol/l. Thus, the concentration of DCA (0.05 mmol/l) was used in the following experiments of the present work. 

***DCA reduced the expression of TLR4 and the production of pro-inflammatory cytokines in LPS-stimulated HTR-8/SVneo cells***

**Figure 3.**
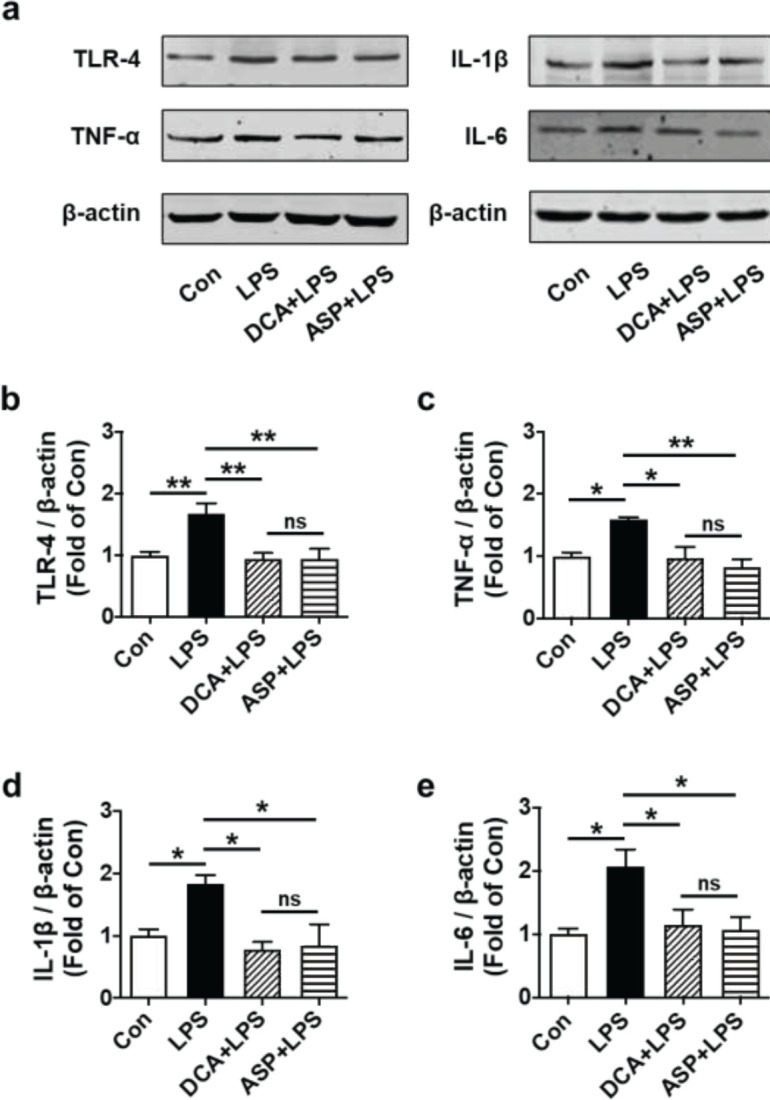
DCA reduced TLR4 and pro-inflammatory cytokines expressions in LPS-stimulated HTR-8/SVneo cells

**Figure 4:**
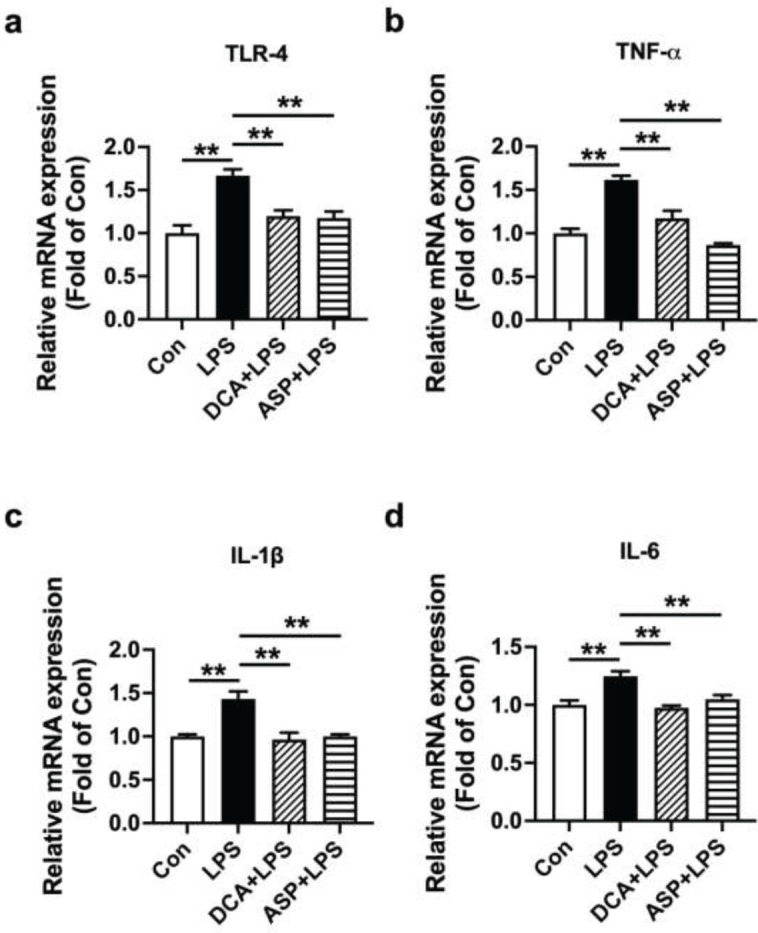
DCA depressed mRNA expression of proinflammatory cytokines in LPS-stimulated HTR-8/SVneo cells

TLR4 is well-known for its function in LPS-induced inflammatory factor production as a direct receptor of LPS. LPS binding to TLR4 mediates the production of the following inflammatory cytokines: IL-1β, TNF-α, and IL-6. HTR-8/SVneo cells were co-treated with LPS (0.5 µg/ml) and DCA (0.05 mmol/l) for 24 hr, then the expression levels of TLR4, TNF-α, IL-1β, and IL-6 were examined through western blotting and RT-PCR. Comparing LPS to the control (+68.2%, **P<0.01; +58.6%, **P<0.01; +84.1%, **P<0.01 and +106.8%, **P<0.01, respectively), there was a substantial rise in the expression of TNF-α, TLR4, IL-6, and IL-1β intracellular levels, while DCA remarkably attenuated these effects (-43.6%, **P<0.01; -38.9%, **P<0.01; -57.5%, **P<0.01; and -44.5%, **P<0.01, respectively) (Figure 3). In addition, DCA significantly reduced TLR4, TNF-α, IL-1β, and IL-6 mRNA levels induced by LPS compared with LPS treatment (**P<0.01) (Figure 4a–d). Meanwhile, treatment with ASP significantly reduced the levels of TLR4, IL-6, IL-1β, and TNF-α mRNA induced by LPS compared with LPS treatment (**P<0.01) (Figure 4a-d). These studies showed that, in LPS-stimulated HTR-8/SVneo cells, DCA reduced the expression of TLR4 and pro-inflammatory cytokines. 

***DCA suppressed LPS-induced phosphorylation of p65 and the translocation of p65 into the nucleus***

**Figure 5:**
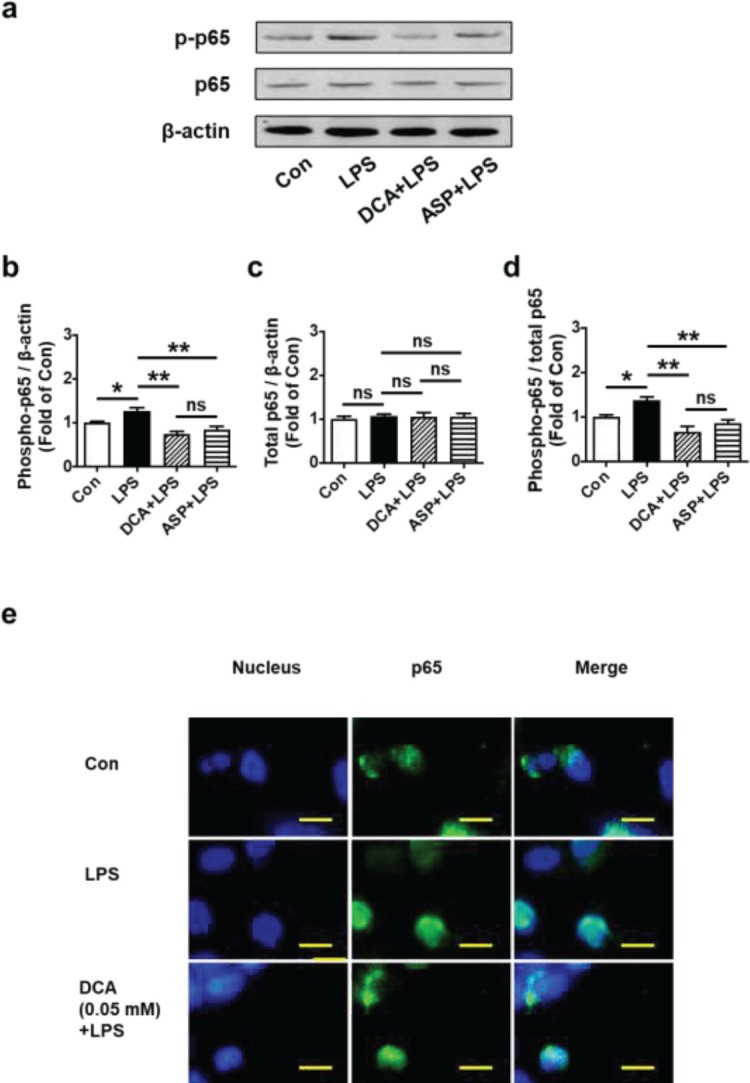
DCA suppressed the phosphorylation and nuclear transfer of NF-κB

It has been suggested that NF-κB played a crucial role in the regulation of pro-inflammatory cytokine production. Downstream of the NF-κB pathway is activated by TLR4 signaling. Previous investigations by others have shown that activation of the NF-κB pathway leads to results in translocation of NF-κB subunits including phospho-p65 to the nucleus (35-37). We investigated whether the NF-κB signaling pathway was responsible for DCA’s ability to reduce inflammatory responses in LPS-stimulated HTR-8/SVneo cells. HTR-8/SVneo cells were co-treated with DCA (0.05 mmol/l) and LPS (0.5 µg/ml) for 24 hr, then the expression levels of phosphorylation of p65 and p65 were detected and examined using blotting. As with cells treated with ASP, we discovered that DCA (0.05 mmol/L) dramatically reduced the phosphorylation of p65 when compared to the control (**P<0.01; Figure 5a-d). We also examined whether the translocation of the p65 subunit of NF-κB from the cell membrane to the nucleus could be disrupted by DCA. NF-κB p65 staining in the nucleus indicated that most intracellular p65 translocated from the cytoplasm to the nucleus in LPS-stimulated cells. By treating cells with DCA (0.5 μg/ml), there was a significant reduction in p65 levels in the nucleus (Figure 5e). 

***DCA inhibited pro-inflammatory cytokine production via TLR4 signaling pathway***

**Figure 6:**
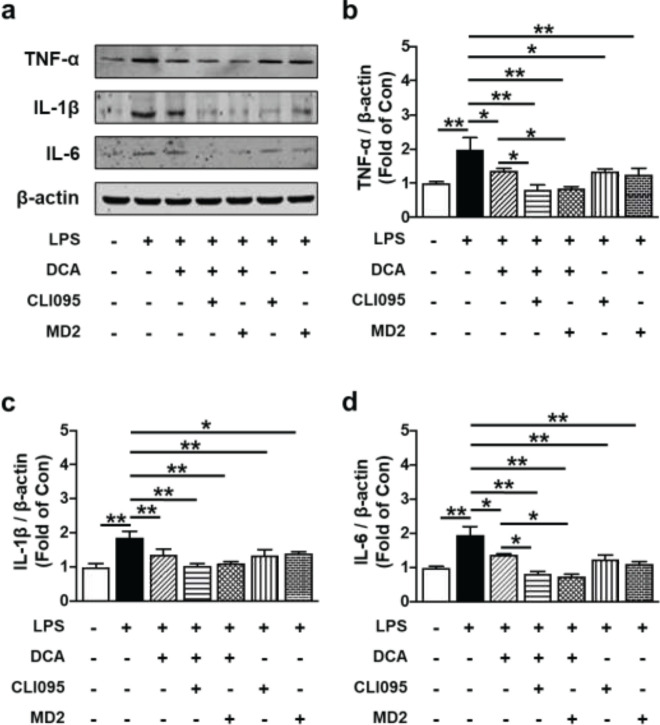
Relationship between DCA’s inhibitory effects on LPS-induced inflammation and TLR4 in HTR-8/SVneo cells

To more thoroughly assess whether DCA suppressed inflammatory responses through TLR4, TLR4 signaling inhibitors were incubated with HTR-8/SVneo cells. Treatment with inhibitors of TLR4 signal transduction (CLI095 and MD2-TLR-4-IN-1) and DCA all suppressed LPS-induced overexpression of TNF-α, IL-1β, and IL-6 in HTR-8/SVneo cells (*P<0.05 and **P<0.01). Additionally, treating HTR-8/SVneo cells with a mix of TLR4 inhibitors and DCA prevented the inflammatory cytokine rise brought on by LPS. (**P<0.01; Figure 6). These findings imply that TLR4 may contribute to DCA’s ability to reduce LPS-induced inflammation in HTR-8/SVneo cells.

## Discussion

In the present study, we found that DCA promoted the migration of LPS-stimulated HTR-8/SVneo cells by suppressing inflammatory response via the TLR4/NF-κB pathway, and the promoting effect of DCA was comparable with that achieved with aspirin (an agent might prevent or delay pre-eclampsia in randomized trials) (38).

The significance of the epithelial-mesenchymal transition (EMT) during the differentiation of extravillous cytotrophoblasts (evCTB) is well established ( 39 ). Epithelial cells undergo a cellular process called EMT that transforms them into mesenchymal cells, enabling them to migrate and metastasize ( 39 ). EMT has proved to play a major role in both early and late development of trophoblasts ( 40 ). The HTR-8/SVneo cell line is a heterogeneous population of trophoblast and stromal cells (41). As a result of further investigations, it was identified that the heterogeneity of the cells was mainly attributed to the ongoing EMT process *in vitro*, which resembles the EMT that occurs during normal trophoblast development (40). Moreover, across different placental cell lines, HTR-8/SVneo is still the one that has been most frequently used to study the invasion, migration, and proliferation of evCTB, a cell line that develops from first-trimester evCTB infected with retroviral vectors (40). 

Clinically, DCA is used to treat lactic academia, including mitochondrial encephalomyopathy and pyruvate dehydrogenase complex deficiency (13). There have been 40 years of human experience with mechanistic investigations of DCA in human tissues following oral administration, and it has been demonstrated that DCA is powerful, long-lasting, and safe in both children and adults (14). The present study demonstrated that DCA could significantly improve the migration function of LPS-stimulated trophoblast cells. Previous studies have demonstrated that impaired trophoblast cell function impeded spiral artery transformation, which may result in pre-eclampsia (6, 7). Accordingly, DCA might have a therapeutic potential to prevent pregnancy complications with trophoblast disorder, such as pre-eclampsia, which remains to be validated in future studies.

Recently, it has been shown that DCA exhibits an anti-inflammatory effect (15-17). DCA was found to alleviate the development of arthritis in female mice in an estrogen-dependent manner and affect macrophage migration function and inflammatory responses by inhibiting glycolytic reprogramming (15, 16). Moreover, there is growing evidence that TLR4 has a critical effect during the activation of innate immune responses as a result of its pattern recognition ability (21). Several models and cell lines have been shown to respond to LPS by coordinating the expression of cytokines and other immune-related genes through TLR4 activation (19-20, 22). In the present work, it was demonstrated that DCA obviously inhibited LPS-induced overexpression of TNF-α, TLR4, IL-6, and IL-1β in HTR-8/SVneo cells. Moreover, we found that treatment with TLR4 inhibitors (CLI095 or MD2-TLR-4-IN-1) reduced overexpression of pro-inflammatory cytokines by LPS, and a synergistic effect was found between TLR4 inhibitors and DCA in HTR-8/SVneo cells (42-45). From this, we speculated that the TLR4 signaling complex may be engaged in the mechanism by the DCA to inhibit LPS-induced inflammation in HTR-8/SVneo cells.

After TLR signaling complex activation at the plasma membrane, overexpression of pro-inflammatory mediators by transcription factors is triggered by the activation of a cascade of intracellular proteins (22). As the prototypical transcription factor, NF-κB has a pivotal role in the innate immune response. The expression level of NF-κB was reported to be obviously elevated in preeclamptic placentas in comparison with normal placentas (46). NF-κB can increase the expression of a diverse range of enzymes and pro-inflammatory cytokines by interacting with DNA and regulating the transcription of target genes, only if NF-κB is activated and translocated into the nucleus (21, 23, 35-37). In this study, we found that DCA can markedly depress the phosphorylation of NF-κB (p65) and the translocation of p65 into the nucleus in HTR-8/SVneo cells stimulated by LPS. This finding suggests that DCA may regulate the LPS-induced inflammatory response in HTR-8/SVneo cells via the NF-κB pathway.

(a) Effect of LPS on cell viability by CCK-8 assay in HTR-8/SVneo cells at 24 hr. n = 6. (b) HTR-8/SVneo cells were treated with LPS (0.1, 0.2, 0.5, or 1 µg/ml) for 24 hr. Then the migration distance of cells was evaluated. Representative images of cell migration by scratch-wound healing assay (up). Scale bar: 200 μm. Quantification of wound repair at 0 and 24 hr after wound scratch in wound-healing assay (below). n = 8-11. LPS: Lipopolysaccharides. Data are presented as means±SEM. *P<0.05, **P<0.01.

(a) Cell migration was detected by scratch wound healing assay. HTR-8/SVneo cells were co-incubated with LPS (0.5 μg/ml) and DCA (0.01, 0.05, and 0.25 mmol/l) or ASP (10 μg/ml) for 24 hr. Then the migration distance of cells was evaluated. Representative images of cell migration by scratch-wound healing assay (up). Scale bar: 200 μm. Quantification of wound repair at 0 and 24 hr after wound scratch in wound-healing assay (below). n = 6-12. (b) Cell migration was detected by transwell migration assay. HTR-8/SVneo cells were co-incubated with LPS (0.5 μg/ml) and DCA (0.05 mmol/l) or APS (10 μg/ml) for 24 hr. Then the migration number of cells was evaluated. Representative images of the migrated cells (left). Scale bar: 200 μm. Quantification of migrated cells at 24 hr (right). n = 6-12. DCA: Sodium dichloroacetate; ASP: Aspirin; LPS: Lipopolysaccharides. Data are presented as means±SEM. *P<0.05, **P<0.01.

Cells were co-incubated with LPS (0.5 μg/ml) and DCA (0.05 mmol/l) or ASP (10 μg/ml) for 24 hr. (a) Representative images of western blotting analysis of TLR4, TNF-α, IL-1β, IL-6, and β-actin proteins in HTR-8/SVneo cells. (b) Expression of TLR4 relative to β-actin. n = 3-5 (c) Expression of TNF-α relative to β-actin. n = 4. (d) Expression of IL-1β relative to β-actin. n = 4. (e) Expression of IL-6 relative to β-actin. n = 5. Values were normalized to Control. DCA: Sodium dichloroacetate; ASP: Aspirin; LPS: Lipopolysaccharides. Data are presented as means±SEM. *P<0.05, **P<0.01.

Cells were co-incubated with LPS (0.5 μg/ml) and DCA (0.05 mmol/L) or ASP (10 μg/ml) for 24 hr. Total RNA was extracted from the HTR-8/SVneo cells and quantitative RT-PCR analysis was performed. Bar graphs show relative mRNA expression of TLR-4 (a), TNF-α (b), IL-1β (c), and IL-6 (d). n=6. RT-PCR, reverse transcription PCR. Data are presented as means±SEM. **P<0.01.

Cells were co-incubated with LPS (0.5 μg/ml) and DCA (0.05 mmol/l) for 24 hr. (a) Representative images of western blotting analysis of NF-κB (p65), phospho-NF-κB p65 (p-p65), and β-actin in HTR-8/SVneo cells. (b-d) Expression of p-p65, p65 relative to β-actin, and p-p65 relative to p65. n = 4–5. (e) Nuclear translocation of nuclear factor-κB (NF-κB) p65 subunit was detected using immunofluorescence assay. Scale bar: 20 µm. Blue and green fluorescence indicates localization of the nucleus (Hoechst) and p65, respectively. The immunostained cells were analyzed with a fluorescence microscope (40 ×). LPS: Lipopolysaccharides; DCA: Sodium dichloroacetate; ASP: Aspirin. Data are presented as means±SEM. *P<0.05, **P<0.01.

HTR-8/Svneo cells were pretreated with or without TLR4 inhibitors (CLI095, 10 µmol/L; MD2-TLR-4-IN-1, 10 µmol/l) for 1 hr before co-incubation with LPS (0.5 μg/ml) and/or DCA (0.05 mmol/l) for 24 hr, then cells were collected for western blotting. (a) Representative images of western blotting analysis of TNF-α, IL-1β, IL-6, and β-actin in HTR-8/SVneo cells. (b-d) Expression of TNF-α, IL-1β, and IL-6 relative to β-actin. n=3. LPS: Lipopolysaccharides; DCA: Sodium dichloroacetate; MD2: MD2-TLR-4-IN-1. Data are presented as mean±SEM. *P<0.05, **P<0.01

**Figure 7:**
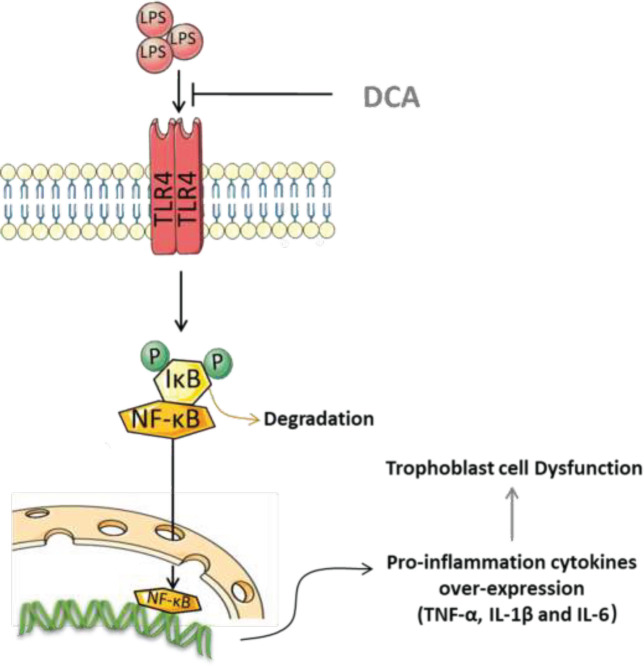
Putative mechanisms underlying DCA improving migration ability in HTR-8/SVneo cells

LPS-induced inflammation in HTR-8/SVneo cells causes cell migration impairment. DCA interacts with TLR4 to modulate pro-inflammatory cytokine activation, therefore improving cell migration ability. DCA: Sodium dichloroacetate

## Conclusion

It was first shown that DCA improved trophoblast cell migration function by suppressing LPS-induced inflammation, at least partially via TLR4/NF-κB signaling pathway.  (Figure 7). This result indicates that DCA might be a potential therapeutic candidate for human pregnancy-related complications associated with trophoblast disorder.

## Authors’ Contributions

HH X conceived and designed the experiments. C L, ZW Z, Y J, JLY, JB H, J L, X T, C Z, CFX. and C P performed the experiments. HH X, X T, C L, ZW Z, Y J, and C P analyzed the data. HH X, C L, Y J, and C P wrote the manuscript.

## Conflicts of Interest

The authors declare that the research was conducted in the absence of any commercial or financial relationships that could be construed as potential conflicts of interest.

## References

[1] Amaral LM, Cornelius DC, Harmon A, Moseley J, Martin JN Jr, LaMarca B (2015). 17-hydroxyprogesterone caproate significantly improves clinical characteristics of PE in the reduced uterine perfusion pressure rat model. Hypertension.

[2] Melchiorre K, Sharma R, Thilaganathan B (2014). Cardiovascular implications in preeclampsia: An overview. Circulation.

[3] Say L, Chou D, Gemmill A, Tunçalp Ö, Moller AB, Daniels J, Gülmezoglu AM, Temmerman M, Alkema L (2014). Global causes of maternal death: A WHO systematic analysis. Lancet Glob Health.

[4] Redman CW, Sacks GP, Sargent IL (1999). Preeclampsia: An excessive maternal inflammatory response to pregnancy. Am J Obstet Gynecol.

[5] Damsky CH, Librach C, Lim KH, Fitzgerald ML, McMaster MT, Janatpour M (1994). Integrin switching regulates normal trophoblast invasion. Development.

[6] Kliman HJ, Nestler JE, Sermasi E, Sanger JM, Strauss JF 3rd (1986). Purification, characterization, and in vitro differentiation of cytotrophoblasts from human term placentae. Endocrinology.

[7] Kohli S, Ranjan S, Hoffmann J, Kashif M, Daniel EA, Al-Dabet MM (2016). Maternal extracellular vesicles and platelets promote preeclampsia via inflammasome activation in trophoblasts. Blood.

[8] Noris M, Perico N, Remuzzi G (2005). Mechanisms of disease: Pre-eclampsia. Nat Clin Pract Nephrol.

[9] Redman CW, Sargent IL (2005). Latest advances in understanding preeclampsia. Science.

[10] Svinarich DM, Bitonti OM, Romero R, Gonik B (1996). Induction and posttranslational expression of cytokines in a first-trimester trophoblast cell line by lipopolysaccharide. Am J Obstet Gynecol.

[11] Zaga-Clavellina V, Garcia-Lopez G, Flores-Herrera H, Espejel-Nuñez A, Flores-Pliego A, Soriano-Becerril (2007). In vitro secretion profiles of interleukin (IL)-1beta, IL-6, IL-8, IL-10, and TNF alpha after selective infection with Escherichia coli in human fetal membranes. Reprod Biol Endocrinol.

[12] Dowling O, Chatterjee PK, Gupta M, Tam Tam HB, Xue X, Lewis D (2012). Magnesium sulfate reduces bacterial LPS-induced inflammation at the maternal-fetal interface. Placenta.

[13] Stacpoole PW (1989). The pharmacology of dichloroacetate. Metabolism.

[14] Michelakis ED, Sutendra G, Dromparis P, Webster L, Haromy A, Niven E et al (2010). Metabolic modulation of glioblastoma with dichloroacetate. Sci Transl Med.

[15] Semba H, Takeda N, Isagawa T, Sugiura Y, Honda K, Wake M et al (2016). HIF-1α-PDK1 axis-induced active glycolysis plays an essential role in macrophage migratory capacity. Nat Commun.

[16] Bian L, Josefsson E, Jonsson IM, Verdrengh M, Ohlsson C, Bokarewa M et al (2009). Dichloroacetate alleviates development of collagen II-induced arthritis in female DBA/1 mice. Arthritis Res Ther.

[17] Horne AW, Ahmad SF, Carter R, Simitsidellis I, Greaves E, Hogg C et al (2019). Repurposing dichloroacetate for the treatment of women with endometriosis. Proc Natl Acad Sci U S A.

[18] Shakhov AN, Collart MA, Vassalli P, Nedospasov SA, Jongeneel CV (1990). Kappa B-type enhancers are involved in lipopolysaccharide-mediated transcriptional activation of the tumor necrosis factor alpha gene in primary macrophages. J Exp Med.

[19] Landoni VI, Schierloh P, de Campos Nebel M, Fernández GC, Calatayud C, Lapponi MJ et al (2012). Shiga toxin 1 induces on lipopolysaccharide-treated astrocytes the release of tumor necrosis factor-alpha that alter brain-like endothelium integrity. PLoS Pathog.

[20] Poltorak A, He X, Smirnova I, Liu MY, Van Huffel C, Du X et al (1998). Defective LPS signaling in C3H/HeJ and C57BL/10ScCr mice: mutations in Tlr4 gene. Science.

[21] Yang X, Haghiac M, Glazebrook P, Minium J, Catalano PM, Hauguel-de Mouzon S (2015). Saturated fatty acids enhance TLR4 immune pathways in human trophoblasts. Hum Reprod..

[22] Cramer M, Nagy I, Murphy BJ, Gassmann M, Hottiger MO, Georgiev O et al (2005). NF-kappaB contributes to transcription of placenta growth factor and interacts with metal responsive transcription factor-1 in hypoxic human cells. Biol Chem.

[23] Logothetou-Rella H, Kotoulas IG, Nesland JM, Kipiotis D, Abazis D (1989). Early human trophoblast cell cultures A morphological and immunocytochemical study. Histol Histopathol.

[24] Chen YY, Xiao L, Sun GQ, Li M, Yang HL, Ming ZY et al (2023). TMBIM4 deficiency facilitates NLRP3 inflammasome activation-induced pyroptosis of trophoblasts: A potential pathogenesis of preeclampsia. Biology (Basel).

[25] Liang CC, Park AY, Guan JL (2007). In vitro scratch assay: A convenient and inexpensive method for analysis of cell migration in vitro. Nat Protoc.

[26] Jonkman JEN, Cathcart JA, Xu F, Bartolini ME, Amon JE, Stevens KM et al (2014). An introduction to the wound healing assay using live-cell microscopy. Cell Adh Migr.

[27] Justus CR, Leffler N, Ruiz-Echevarria M, Yang LV (2014). In vitro cell migration and invasion assays. J Vis Exp.

[28] Abbas Y, Turco MY, Burton GJ, Moffett A (2020). Investigation of human trophoblast invasion in vitro. Hum Reprod Update.

[29] Peng C, Yang LJ, Zhang C, Jiang Y, Shang LLWX, He JB et al (2023). Low-dose nifedipine rescues impaired endothelial progenitor cell-mediated angiogenesis in diabetic mice. Acta Pharmacol Sin.

[30] Otsuka K, Terasaki F, Ikemoto M, Fujita SC, Tsukada B, Katashima T (2009). Suppression of inflammation in rat autoimmune myocarditis by S100A8/A9 through modulation of the proinflammatory cytokine network. Eur J Heart Fail.

[31] Liu KH, Zhang BL, Yang BS, Shi WT, Li YF, Wang Y et al (2021). Rhizobiales-specific RirA represses a naturally “synthetic” foreign siderophore gene cluster to maintain sinorhizobium-legume mutualism. mBio.

[32] Qi D, Lu JY, Fu ZY, Lv SS, Hou LL (2022). Psoralen promotes proliferation, migration, and invasion of human extravillous trophoblast derived HTR-8/Svneo cells in vitro by NF-κB pathway. Front Pharmacol.

[33] Janovec V, Ryabchenko B, Škarková A, Pokorná K, Rösel D, Brábek J et al (2021). TLR4-mediated recognition of mouse polyomavirus promotes cancer-associated fibroblast-like phenotype and cell invasiveness. Cancers (Basel).

[34] Oh KK, Adnan M, Cho DH (2022). Drug investigation to dampen the comorbidity of rheumatoid arthritis and osteoporosis via molecular docking test. Curr Issues Mol Biol.

[35] Zhang YY, Zhang Y, Yao YB, Lei XL, Qian ZJ (2018). Butyrolactone-I from coral-derived fungus aspergillus terreus attenuates neuro-inflammatory response via suppression of NF-κB pathway in BV-2 cells. Mar Drugs.

[36] Du M, Yuan L, Tan X, Huang D, Wang X, Zheng Z et al (2017). The LPS-inducible lncRNA Mirt2 is a negative regulator of inflammation. Nat Commun.

[37] Gao H, Sun W, Zhao J, Wu X, Lu JJ, Chen X et al (2016). Tanshinones and diethyl blechnics with anti-inflammatory and anti-cancer activities from Salvia miltiorrhiza Bunge (Danshen). Sci Rep.

[38] Askie LM, Duley L, Henderson-Smart DJ, Stewart LA (2007). Antiplatelet agents for prevention of pre-eclampsia: A meta-analysis of individual patient data. Lancet.

[39] Davies JE, Pollheimer J, Yong HEJ, Kokkinos MI, Kalionis B, Knöfler M et al (2016). Epithelial-mesenchymal transition during extravillous trophoblast differentiation. Cell Adh Migr.

[40] Msheik H, Azar J, Sabeh ME, Abou-Kheir W, Daoud G (2020). HTR-8/SVneo: A model for epithelial to mesenchymal transition in the human placenta. Placenta.

[41] Abou-Kheir W, Barrak J, Hadadeh O, Daoud G (2017). HTR-8/SVneo cell line contains a mixed population of cells. Placenta.

[42] Granger JP (2004). Inflammatory cytokines, vascular function, and hypertension. Am J Physiol Regul Integr Comp Physiol.

[43] Takashima K, Matsunaga N, Yoshimatsu M, Hazeki K, Kaisho T, Uekata M et al (2009). Analysis of binding site for the novel small-molecule TLR4 signal transduction inhibitor TAK-242 and its therapeutic effect on mouse sepsis model. Br J Pharmacol.

[44] Liu Z, Chen L, Yu P, Zhang Y, Fang B, Wu C et al (2019). Discovery of 3-(Indol-5-yl)-indazole derivatives as novel myeloid differentiation protein 2/Toll-like receptor 4 antagonists for treatment of acute lung injury. J Med Chem.

[45] Shimazu R, Akashi S, Ogata H, Nagai Y, Fukudome K, Miyake K et al (1999). MD-2, a molecule that confers lipopolysaccharide responsiveness on Toll-like receptor 4. J Exp Med.

[46] Vaughan JE, Walsh SW (2012). Activation of NF-κB in placentas of women with preeclampsia. Hypertens Pregnancy.

